# An Optimum Fatigue Design of Polymer Composite Compressed Natural Gas Tank Using Hybrid Finite Element-Response Surface Methods

**DOI:** 10.3390/polym13040483

**Published:** 2021-02-03

**Authors:** Kazem Reza Kashyzadeh, Seyed Saeid Rahimian Koloor, Mostafa Omidi Bidgoli, Michal Petrů, Alireza Amiri Asfarjani

**Affiliations:** 1 Department of Transport, Academy of Engineering, Peoples’ Friendship University of Russia (RUDN University), 6 Miklukho-Maklaya Street, 117198 Moscow, Russia; 2 Department of Mechanical and Instrumental Engineering, Academy of Engineering, Peoples’ Friendship University of Russia (RUDN University), 6 Miklukho-Maklaya Street, 117198 Moscow, Russia; 3 Institute for Nanomaterials, Advanced Technologies and Innovation, Technical University of Liberec, Studentska 2, 461 17 Liberec, Czech Republic; s.s.r.koloor@gmail.com (S.S.R.K.); michal.petru@tul.cz (M.P.); 4 School of Mechanical Engineering, Faculty of Engineering, University Technology Malaysia (UTM), Johor Bahru 81310, Malaysia; 5 Department of Mechanical Engineering, Islamic Azad University, Badroud Branch, Badroud, Isfahan 8764136545, Iran; mostafaomidibidgoli@gmail.com; 6 Department of Mechanical Engineering, Shahrood University of Technology, Shahrood 3619995161, Iran; alirezaamiri20@gmail.com

**Keywords:** gas tanks for vehicles, polymer composite tank, type-4 CNG tank, fatigue life, optimization, response surface analysis, finite element simulation

## Abstract

The main purpose of this research is to design a high-fatigue performance hoop wrapped compressed natural gas (CNG) composite cylinder. To this end, an optimization algorithm was presented as a combination of finite element simulation (FES) and response surface analysis (RSA). The geometrical model was prepared as a variable wall-thickness following the experimental measurements. Next, transient dynamic analysis was performed subjected to the refueling process, including the minimum and maximum internal pressures of 20 and 200 bar, respectively. The time histories of stress tensor components were extracted in the critical region. Furthermore, RSA was utilized to investigate the interaction effects of various polymer composite shell manufacturing process parameters (thickness and fiber angle) on the fatigue life of polymer composite CNG pressure tank (type-4). In the optimization procedure, four parameters including wall-thickness of the composite shell in three different sections of the CNG tank and fiber angle were considered as input variables. In addition, the maximum principal stress of the component was considered as the objective function. Eventually, the fatigue life of the polymer composite tank was calculated using stress-based failure criterion. The results indicated that the proposed new design (applying optimal parameters) leads to improve the fatigue life of the polymer composite tank with polyethylene liner about 2.4 times in comparison with the initial design.

## 1. Introduction

Compressed natural gas (CNG) is used as a good alternative to petrol fuel in land transportation (especially, automotive) in many countries. The CNG fuel tank should be manufactured with a high safety factor and pass stringent standards to get the production license because this super-critical component deals directly with human life. On the other hand, hazards from the failure of CNG automotive cylinders can cause serious damage to the vehicle and even the death of the driver or occupants. CNG fuel tanks were first made of metal. Afterward, the usage of composite materials in the industry has led to the discovery of type-3 composite tanks with the aim of weight reduction. This type of tank consists of a metal liner and a polymer composite shell reinforced with carbon or glass fiber. However, due to the bursting of the metal layer due to cyclic internal pressures (fatigue phenomenon), the modified design of the composite tank uses a non-metallic liner such as polyethylene, nylon, polyether ether ketone, polyethylene terephthalate, and polypropylene (finding the type-4 composite tank). One of the most important advantages of this particular type of CNG fuel tank compared to other types is that fatigue failure appears as the leakage of the gas and as a result prevents the tank from bursting suddenly. It is noteworthy that, given the high sensitivity of this component, little research has been done to investigate the behavior of the polymer composite CNG fuel tank under fatigue loads and various working conditions such as in the vicinity of the fire. However, the details of some of these studies are discussed below.

The behavior of a type-3 composite tank considering the low alloy steel liner has been investigated under static loading (maximum internal pressure) [1]. Their research showed that the use of composite tanks compared to the metal tank has more corrosion resistance and the least stress due to fuel filling. In addition, they stated that the weight of the composite tank is much less than the weight of the metal tank. Moreover, finite element simulation was used to study the failure of the composite type-4 CNG tank [2]. In this research, a two-liter tank has been used, which is very small compared to the tanks used in the automotive industry. However, no experimental test was performed and the published results in other studies were used to validate the presented finite element (FE) model and use of the WCM plugin. Finally, various static failure criteria of composite materials including Tsai–Hill, Tsai–Wu, and Hashin have been applied and burst pressure has been calculated. Finally, they reported that the damage rate and breakdown in the spiral layers of the tank are higher than the peripheral layers; also, the regularity of the layer destruction is different.

The cyclic lifetime of the CNG composite tank with the surface defects (notch with the same width and depth, which is considered to be equal to 0.2 wall-thickness) has been predicted by utilizing the strain-life criterion [3]. Kim and Choi have performed a risk analysis of the CNG composite tank to determine the period time of visual inspection [4]. The findings of this research stated that the status of gas leakage, internal pressure, and temperature should be checked every three years.

Nouri et al. have estimated the static strength of the CNG polymer composite cylinder tank subjected to the maximum level of internal pressure [5]. They used different composite failure theories under static loading. By comparing the obtained results with laboratory data, they stated that Hoffmann benchmarking gave the most accurate prediction of static strength. However, due to its complex calculations, it is recommended to use Tsai–Wu, Tsai–Hill, and Christensen criteria to reduce computational costs. Also, the results of this study showed that the shear stress in the XY plane is the main cause of tank failure. Moreover, they reported that the separation between the composite shell and the lower aluminum flange occurs.

Seyedi et al. have analyzed and optimized the type-4 composite fuel tanks (without metal) [6]. In the first step, the stress analysis of a metallic tank (made of carbon-manganese) was performed in finite element software. Then, the prediction of fracture zone was validated by comparison with experimental work. Next, the polymer composite tank was examined. One of the weaknesses of this study was considering the the whole wall of the tank to be a constant thickness. In addition, the geometric modeling was based on the sizes in the standard, which will be different from the actual measurements, due to the different stages of manufacturing. Also, the arrangement of the layers is considered to be 45°. Finally, to optimize the strength and weight of the tank, four variables including the thickness of the liner and the composite shell, and the pressure inside the tank are considered. But the fiber angle is assumed to be constant in the optimization process. Also, to optimize, no special technique has been used, and only for each of the parameters, a range including low and high limits and interval has been selected and the results were compared with each other by trial and error. In this study, they showed that by maintaining the static strength of the tank, they can reduce its weight by more than half.

Reynaldo et al. studied the performance of a type-4 pressure vessel as a fuel tank to store the liquid ammonia [7]. They analyzed different polymer materials that are compatible with this type of fuel (e.g., high-density polyethylene, nylon, polyether ether ketone, polyethylene terephthalate, and polypropylene). Also, two fiber-reinforced polymers including carbon and glass were investigated. They reported that the best structure for this tank is the combination of CFRP and PET as a liner. Of course, this result is obtained only concerning the impact condition. In this research, it is assumed that the designed tank has a fixed wall-thickness and the layout was [90°, θ], in which θ = 10°, 30°, 50°, and 70°. In this area, other studies have been conducted to design, and perform stress analysis, failure analysis, structural stability, and optimization to store hydrogen as a vehicle fuel [8,9]. In addition, the safety analysis of the instantaneous release of CNG from a tank was investigated [10]. Altuwair and Khan reported that the probability of the damage increases with decreasing mechanical energy coefficient and as a consequence, the explosion rate rises. 

Tschirschwitz et al. have experimentally studied the hazards from the failure of CNG automotive cylinders in the fire [11]. In this research, various types of polymer composite tanks (type-3 and type-4) have been tested. An assessment of fire safety risks was done for the CNG bus systems [12] and the mean fire fatality risk was obtained to be about 0.23 per 100-million miles. Yersak et al. have studied the thermal gradient and the temperature of the polymer composite tank due to the fast process of refilling [13]. The experimental results showed that for a 18.2 s refill, there is a 23.9 °C m^−1^ longitudinal thermal gradient and a 125.6 °C gas temperature. Moreover, Dicken and Merida have experimentally evaluated the influence of filling time on temperature distribution [14]. They reported that the final gas temperature decreases by raising the time of the filling process. In this regard, some studies have been performed using CFD simulation [15,16] and experimental setup [17,18].

Despite all the results of the researchers’ studies, the automotive industry is still facing the explosion of CNG fuel tanks, causing personal and financial damage to individuals. One of the most important reasons for the inconsistency of research results with reality is the use of a simplified model (for example, a perfectly symmetrical geometric model, with constant wall-thickness, etc.). Moreover, ignoring the actual performance of the tank during its service life and not performing analysis following reality (using static and quasi-static analysis instead of the dynamic transient with time under the pretext of cost and a long time to solve problems). Therefore, the response of optimization analyzes based on these principles cannot significantly reduce the occurrence of these events in the world. In the present research, the cyclic behavior of a type-4 CNG fuel tank considering variable wall-thickness of the polymer composite shell was investigated using finite element simulation. Moreover, for the first time, the response surface method as a well-known data mining technique was used to optimize the manufacturing process parameters including fiber angle and wall-thickness in different parts of the structure.

## 2. Methodology

**Figure 1:**
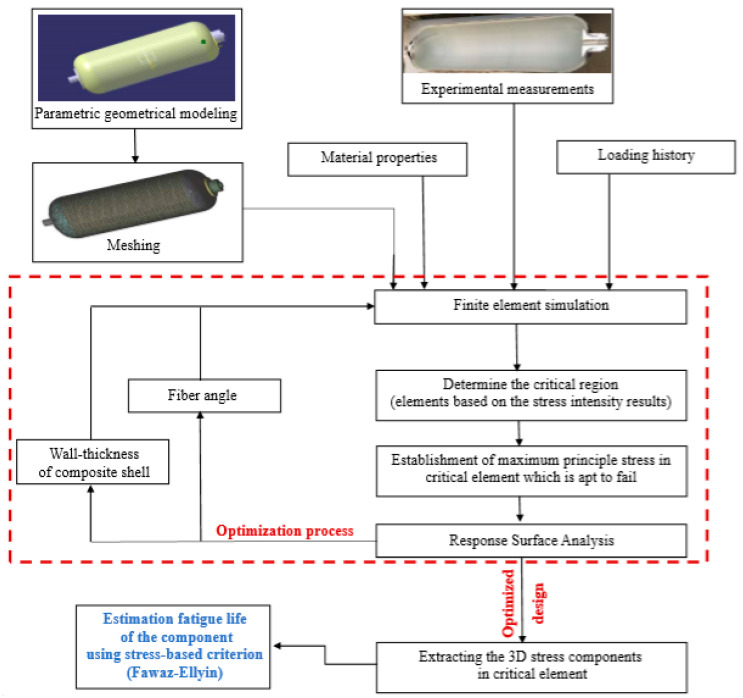
The proposed algorithm for fatigue life improvement of a composite type-4 compressed natural gas (CNG) tank.

As it is clear from the literature review, several parameters are important and effective in analyzing the stress of a CNG polymer composite tank and examining its strength and efficiency. One of them is the thickness of the composite shell, which varies in the cylindrical fuel tank. Its geometry includes different parts such as the cylinder in the main body, the hemisphere in the head of the tank, and also the connection of these two geometries to each other. In many studies done in the past, this parameter was considered constant and in the present research, in addition to modeling, it varies according to reality; this parameter was optimized in different parts of the CNG polymer composite tank. Another important parameter is the fiber angle and the layout of the composite, which the vast majority of researchers have considered in their studies and tried to increase the strength of the tank by choosing the most appropriate fiber angle. In this study, this factor was considered as a variable in the optimization process. Moreover, one of the advantages of this research compared to the others is the objective function in the optimization process. As in previous research, weight reduction or increase in static strength of the fuel tank has been considered. But, in this study, the service life of the fuel tank under fatigue loading due to the refueling process was considered. The flowchart of the process of getting the longest cyclic lifetime of the polymer composite tank by optimizing various composite shell manufacturing process parameters including fiber angle and wall-thickness is summarized in Figure 1.

In the proposed algorithm, firstly the geometric model of the tank is provided as a parametric model using the empirical measurements [5]. Then, finite element simulation of the problem is done using the geometric model, meshing process, applying the boundary conditions and load history, and defining the material properties [5]. Based on the finite element (FE) results, the critical area is determined using maximum principal stress [19]. Next, the optimization process was performed using the response surface method considering the most effective parameter [20,21,22]. After reaching the optimum design that results in the least principal stress, the fatigue life of the component was calculated by employing one of the well-known stress-based failure criteria for polymer composite materials.

## 3. Finite Element Simulation

**Figure 2:**
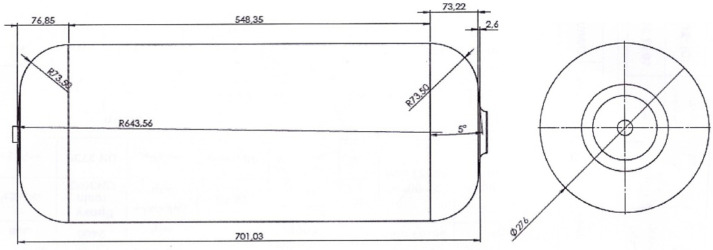
Schematic of the CNG tank with all dimensions in mm [5].

**Figure 3:**
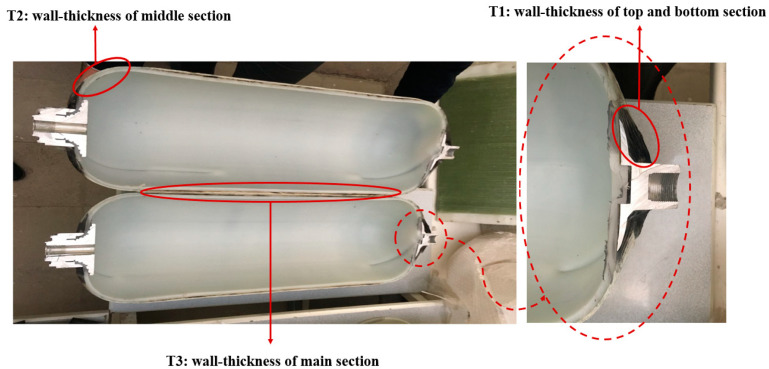
The naming of different parts of the composite shell based on the wall-thickness in a sheared tank.

In the present research, a case study was conducted on the 34-L CNG tank for application in light passenger vehicles in an Asian country (Iran). The dimensions and size of the CNG tank are shown in Figure 2. The CATIA software (Dassault Systèmes SE, Vélizy-Villacoublay, France) was used to model the geometry of the tank as having a variable wall-thickness following the experimental measurements. As shown in Figure 3, the liner thickness is uniform through all parts of the tank (7 mm). However, composite shell thickness is variable (minimum thickness is related to the middle part of the tank and it is maximum at the beginning and end of the tank).

**Figure 4:**
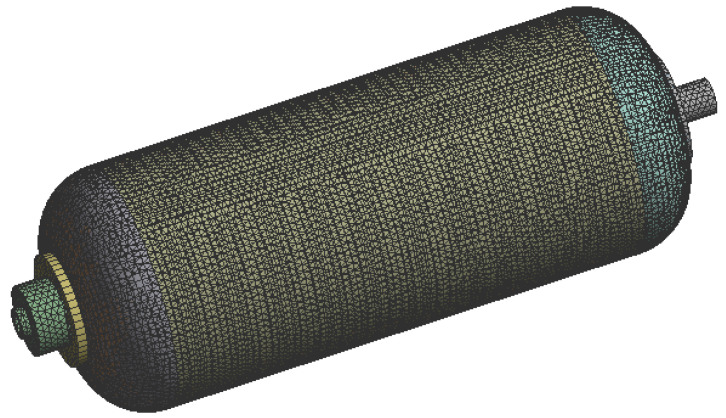
Finite element model of the CNG fuel tank with 91,622 elements [5].

This model includes shell (polymer composite) and volumetric (liner and flanges) parts [5]. Hence, two types of elements (shell and solid) were used to mesh the component in the Altair Hypermesh (Figure 4). To increase the accuracy of the solution and reliable response, a 2nd order element with mid-side nodes was used for meshing composite layers and liner. Moreover, the adhesion between different parts (composite shell, liner, and flanges) was considered 100% [5], and the bonded type of contact was defined. However, for meshing aluminum flanges, the first order solid element was used. Also, a valid technique was applied to couple the shell-to-solid elements [23]. The convergence mesh was performed to select an acceptable element size; then further mesh refinement does not significantly affect the resulting maximum principal stress of the tank [19,23,24].

This model of the CNG tank (Type-4) consists of three main parts called: flanges, liner, and polymer composite shell. Therefore, three different materials (aluminum alloy, polyethylene, and glass/epoxy composite, respectively) were defined in the ANSYS WORKBENCH (Ansys Inc., Canonsburg, PA, USA). Mechanical properties of orthotropic and homogeneous materials are presented in Table 1 and Table 2, respectively.

In the manufacturing process of this tank in the industry, the fiber of Glass 2400, Epoxy resin UN3082, and UN 2735 hardener are used, considering a fiber volume fraction of 50%. In addition, the thickness of the layers is constant and equal to 0.55 mm. The static properties required for simulation of each composite layer can be calculated using three-point bending results such as stiffness, Young’s modulus of bending, flexural rigidity, load, and deflection at maximum and breakpoint [25]. Moreover, the fiber angle of ±17° was considered as the tank manufacturer’s recommendation.

**Figure 5:**
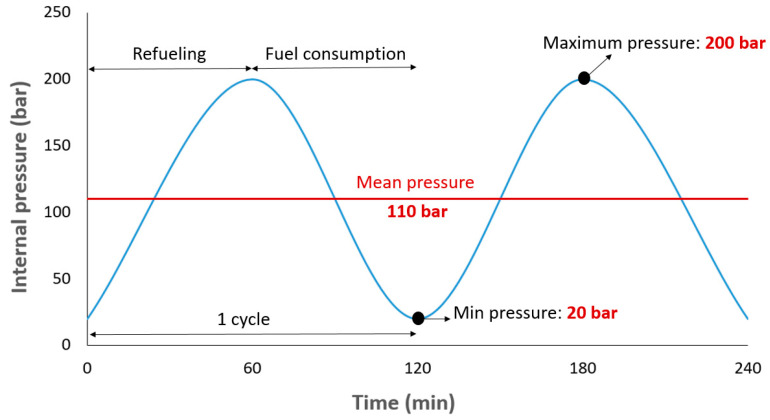
Details of the cyclic loading used to predict the fatigue life of the CNG fuel tank.

In the present FE model, there is no separation between the different parts of the tank during its service life. The internal pressure of 20–200 bar (given equal time for the fill and empty processes) was applied on the full model of the CNG tank and both sides of the tank including flanges that were fixed in all directions. The loading diagram is displayed in Figure 5.

## 4. Response Surface Method

**Figure 6:**
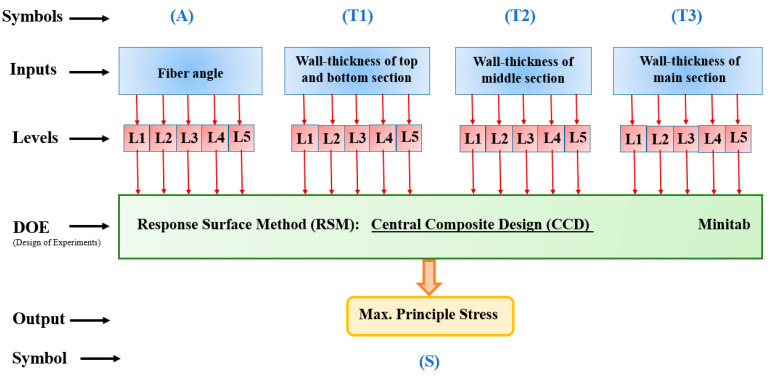
The schematic of the response surface method (RSM)-based design of experiments used in this study.

Various designs of experiment techniques (DOEs) are used to investigate the effect of different parameters on the response when the number of samples is too large and we do not want to examine all of them. In other words, by performing some experiments with predefined settings, the effect of different parameters on the response can be achieved with high accuracy. In this regard, the Taguchi approach represents the minimum number of experiments [20,21,22,24]. But this method can only examine the effect of each parameter on the response separately. However, the response surface method can investigate the effects of changes in two parameters simultaneously on the response. In other words, one of the advantages of using the response surface method (RSM) compared to other data mining methods and design of experiment (DOE) techniques is examining the effect of parameters on the response by considering the interaction between them. Moreover, the output of this method is three-dimensional diagrams including changes of two independent parameters and the response that can be used to optimize the problem. Therefore, this method was used to more accurately investigate the effect of different parameters of the composite tank manufacturing process on the service time of the tank and finally the optimization of the parameters to increase the efficiency of the tank. To this end, four parameters including wall-thickness of polymer composite shell in three different sections of CNG tank and fiber angle were considered as input variables. In addition, five levels were considered for each input variable. The maximum principal stress of the component was considered as an output. A schematic of the used algorithm including the input variables and response is illustrated in Figure 6. Moreover, the variables and different levels considered as inputs for the response surface analysis are given in Table 3. In this study, the linear effects of each parameter and the interaction between them were considered as follows:

A, T1, T2, T3, AT1, AT2, AT3, T1T2, T1T3, and T2T3.

After analyzing the data by RSM [20,21,22] to investigate the effect of parameters (wall-thickness of composite shell in three different sections of the CNG tank and the fiber angle) on the response (maximum principle stress), the optimization tool was used to reduce stress in the critical region of the structure and the optimum values for the parameters were extracted. Since there is a direct relationship between the fatigue life of the component and stress, the optimum tank is expected to have a maximum fatigue life.

## 5. Results and Discussion

**Figure 7:**
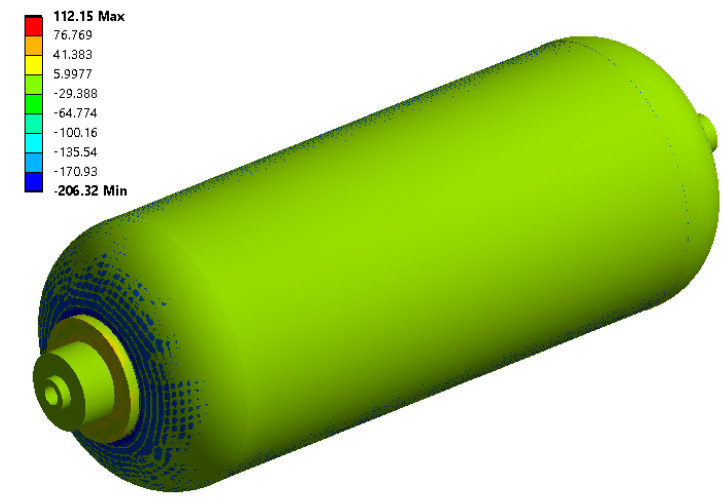
The contour of principal stress in the initial design of the CNG tank.

Explicit transient dynamics analysis was performed and the stress tensor components were extracted in terms of time. The small time increments were considered for the convergence of the solution, then, the time history of principal stress was obtained as the FE response [26,27,28] for all cases proposed by RSM. Next, the maximum value in each history was considered as the output in the response surface method (Table 4). To facilitate the analysis by the response surface method, the response values are always considered positive [29]. Also, the principal stress contour in the CNG tank concerning the values of parameters provided by the manufacturer (initial design) is demonstrated in Figure 7. According to the results of the FE analysis, the highest absolute principle stress (S = 206.32 MPa) is related to the joint area of the composite shell to the flange, which is consistent with the results presented by Nouri et al. [5]. Then, the bonding area between the composite layers in the two parts of the main body and the spherical curvature is critical. Therefore, these two regions in the composite CNG tank are identified as critical areas that are apt to fail and future analysis will be carried out to estimate fatigue life only in the critical elements of these two areas.

**Figure 8:**
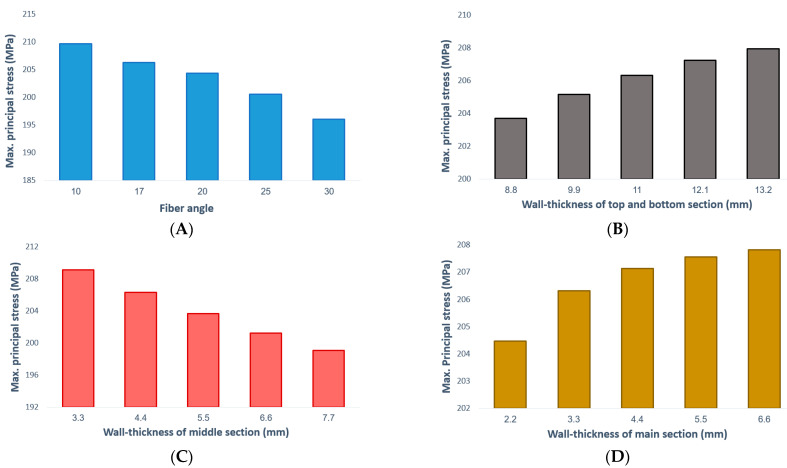
Maximum principal stress created in the critical area of the composite CNG tank in terms of various parameters including (**A**) variation of the fiber angle and the assumption T1 = 11 mm, T2 = 4.4 mm, and T3 = 3.3 mm, (**B**) variation of the wall-thickness of the top and bottom sections and the assumption that the fiber angle = 17, T2 = 4.4 mm, and T3 = 3.3 mm, (**C**) variation of the wall-thickness of the middle section and the assumption that the fiber angle = 17, T1 = 11 mm, and T3 = 3.3 mm, and (**D**) variation of the wall-thickness of the main section and the assumption that the fiber angle = 17, T1 = 11 mm, and T2 = 4.4 mm.

The design of the CNG tanks is based on high-cycle fatigue (HCF) life. In this type of fatigue, the deformation occurs in the elastic region. Hence, stress-based criteria are considered. Finally, it can be stated that the value of stress created in the component is directly related to its fatigue life and the damage rate. As the stress level decreases, the fatigue life of the component increases. The stress created in the composite shell depends on the wall-thickness. On the other hand, it is clear that by changing the angle of the fibers, the directions of the principal axes also change and the stress created is also affected. Figure 8 presents the effect of each parameter on the stress created in the critical area of the composite shell.

From Figure 8, increasing the fiber angle reduces the stress level in the composite shell. Therefore, it seems better to use the maximum twisting angle of fibers in the manufacturing process. Moreover, simulations showed that increasing the wall-thickness in different parts has different results on the stress level. Increasing the wall-thickness of the middle section leads to a decrease in the level of stress, and conversely, by increasing the wall-thickness of the other parts, the level of stress increases. Therefore, to achieve an optimal design in composite CNG fuel tanks, the thickness of the composite shell should be considered and an intermediate value should be determined for them so that behavioral improvements in one area do not lead to damage to another.

**Figure 9:**
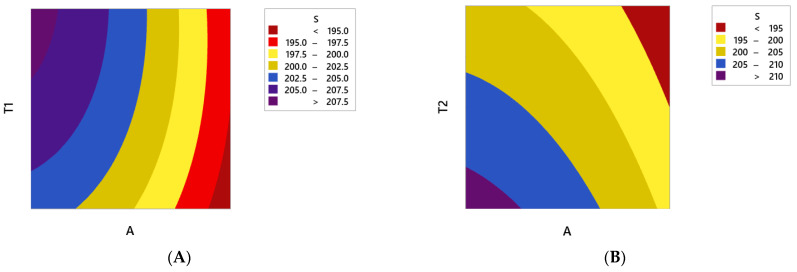

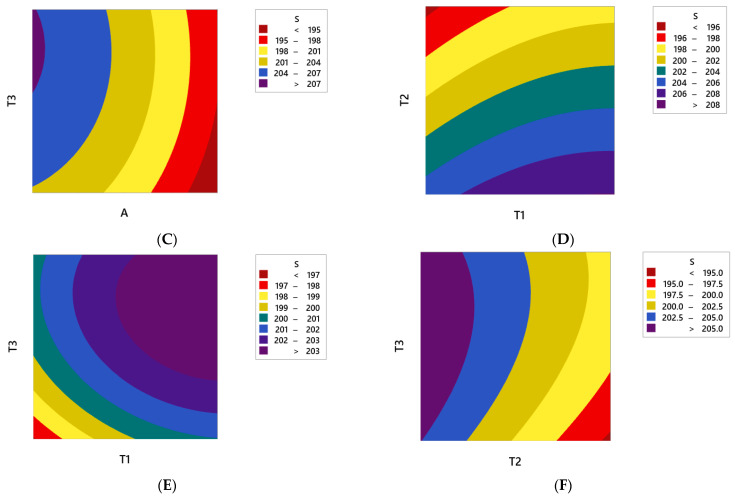
RSM results for maximum principal stress in a variable wall-thickness composite CNG tank including (**A**) variations of the fiber angle and the wall-thickness of the top and bottom sections, (**B**) variations of the fiber angle and the wall-thickness of the middle section, (**C**) variations of the fiber angle and the wall-thickness of the main section, (**D**) variations of the wall-thickness of the top and bottom sections and the wall-thickness of the middle section, (**E**) variations of the wall-thickness of the top and bottom sections and the wall-thickness of the main section, and (**F**) variations of the wall-thickness of the middle section and the wall-thickness of the main section (all symbols are defined in Table 3).

Next, the results of the RSM analysis are illustrated in Figure 9. It should be noted that in these analyses, the constant parameters were assumed to be the average level (L3) [20,21,22].

**Figure 10:**
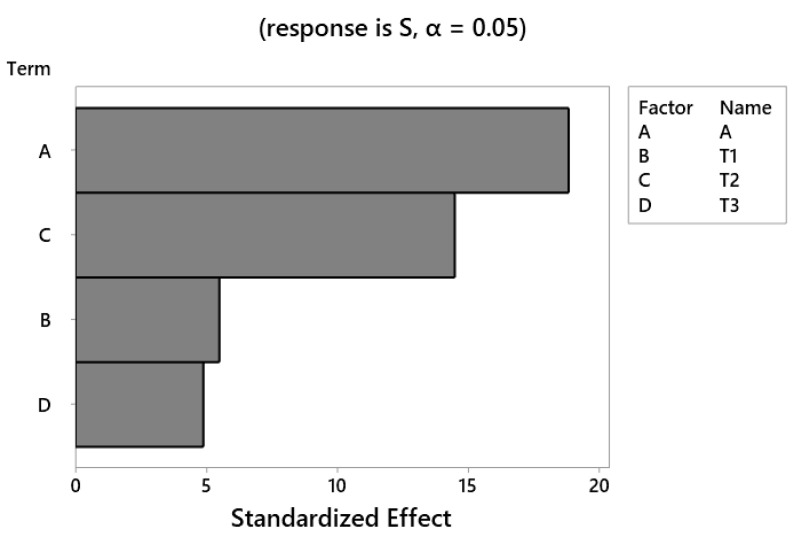
Pareto chart of the standardized effects.

Moreover, a Pareto chart of the standardized effects was used to determine the most effective parameter in the system response. As shown in Figure 10, the parameter of fiber angle has the greatest effect on the performance of composite CNG tanks; also among the thickness parameters, wall-thickness in the middle section is most important. The least effective parameter on the stress results is the wall-thickness of the cylindrical part of the tank, because the main section has a more uniform structure than the other sections as well as low-stress concentration. These results are completely consistent with the simulation results described above (Figure 8). Eventually, the most important results extracted from the contours of Figure 9 are:

1. The maximum principal stress always decreases by increasing the fiber angle. Therefore, it is recommended to consider the maximum level of fiber angle to achieve the maximum fatigue lifetime of the polymer composite CNG tank.
2. In order to have the minimum principal stress in the polymer composite CNG tank, it is necessary to consider the maximum and minimum level settings for parameters of T2 and T1, respectively. On the other hand, it is better to make the composite shell thickness as uniform as possible in the two curved sections of the CNG tank.

In the following, the optimization process was performed using RSM and the values were compared with the baseline design data in Table 5. The findings of this research reveal that the stress level in the optimum composite CNG tank decreases by 10.12%.

In the last step, the fatigue life analysis in the time domain was carried out by using coupling results of stress analyses under cyclic loading (internal pressure of 20 to 200 Bar) and Fawaz–Ellyin criterion [30] in the NCODE DESIGN LIFE software [28]. The obtained results indicate that the fatigue life of the optimized CNG tank is 218,687 cycles. However, the cyclic lifetime for the initial design was 89,841 cycles. In other words, the service life of the variable wall-thickness composite CNG tank can be extended about 2.4 times by utilizing the proposed optimum design.

## 6. Conclusions

In the present research, the authors have attempted to extend the service life of the CNG fuel tank. To achieve this purpose, the study was performed on a real tank sample with variable wall-thickness in different parts. A hybrid finite element-response surface method was presented to predict the strength of polymer composite CNG tanks via wall thickness and fiber angle. Eventually, these two parameters were optimized using the RSM, and the fatigue life of the optimal tank was compared with the original tank. The following practical hints may be deduced from the presented results in this study:

1. The FE results indicate that the maximum principal stress created in the initial design of the CNG composite tank (A = 17, T1 = 11 mm, T2 = 4.4 mm, and T3 = 3.3 mm) under static load is about 206 MPa. Also, the fatigue life of the tank under variations of internal pressure (20–200 bar) is 89,841 cycles. It means that the number of refueling times without the risk of tank failure is equal to 89,841.
2. The Pareto chart as a result of response surface analysis reveals that the most and least effective parameters on the stress level of the CNG composite tank are fiber angle and wall-thickness of the main section, respectively. Therefore, the authors strongly recommend to the manufacturers of Type-4 CNG fuel tanks to be more careful about the twist angle of the fibers in the manufacturing process to strengthen this super-critical component.
3. The RSM results show that the maximum principal stress always decreases by increasing fiber angle. Therefore, it is recommended to consider the maximum level of the fiber angle to achieve the maximum fatigue lifetime of the composite CNG tank. Moreover, to have the minimum principal stress in the polymer composite CNG tank, it is necessary to consider the maximum and minimum level settings for parameters T2 and T1, respectively. On the other hand, it is better to make the thickness of the polymer composite shell as uniform as possible in the two curved sections of the CNG tank. In other words, to achieve a longer service life, it is necessary to try to build tanks with invariable thickness.
4. Based on the optimization algorithm presented, the optimal parameters obtained for the tank are A = 30, T1 = 8.8 mm, T2 = 7.7 mm, and T3 = 2.2 mm. Also, the maximum principal stress and fatigue life related to the optimized tank are obtained about 185 MPa and 218,678 cycles, respectively.
5. The findings of this research state that the maximum principal stress decreases by up to 10% and after that the fatigue life of the fuel tank increases about 2.4 times using optimal values of manufacturing process parameters. After confirmation of the results by pre-production tests, it can drastically reduce the financial losses and irreparable damages due to the fuel tank explosion.

## Figures and Tables

**Table 1:** Mechanical properties of orthotropic materials [5].

Parameter	Symbol	Unit	Polyethylene	Value Glass/Epoxy Composite
Longitudinal tension modulus	*E*_11_	GPa	0.9	35
Transverse tension modulus	*E*_22_	GPa	0.9	3.5
Shear modulus	*G*_12_	GPa	0.312	4.6
Poisson’s ratio	*ϑ*_12_		0.45	0.26

**Table 2:** Mechanical properties of 7xxx series aluminum alloy (Al 7075-T6) [5].

Parameter	Symbol	Unit	Value
Tensile ultimate strength	*σ_ut_*	MPa	572
Tensile yield strength	*σ_yt_*	MPa	503
Elastic modulus	*E*	GPa	71.7
Shear modulus	*G*	GPa	26.9
Poisson’s ratio	*ϑ*		0.33
Fatigue limit	*σ_f_*	MPa	159

**Table 3:** Variables and their levels are used as input data in the response surface method (RSM).

		Levels				
Parameters	Symbol	L1	L2	L3	L4	L5
Fiber angle	A	10	17	20	25	30
Wall-thickness of the top and bottom sections	T1	8.8	9.9	11	12.1	13.2
Wall-thickness of the middle section	T2	3.3	4.4	5.5	6.6	7.7
Wall-thickness of the main section	T3	2.2	3.3	4.4	5.5	6.6

**Table 4:** Finite element results for the different settings of wall-thickness parameters and fiber angle based on the RSM.

Simulation No.	Level of Input Parameters				Output (MPa)
	A	T1	T2	T3	
1	L5	L3	L3	L3	194.84
2	L3	L3	L3	L3	202.77
3	L2	L4	L4	L4	203.68
4	L4	L4	L2	L2	201.27
5	L3	L1	L3	L3	200.35
6	L4	L4	L4	L2	196.51
7	L4	L2	L4	L4	196.78
8	L2	L4	L4	L2	202.06
9	L3	L3	L3	L1	199.75
10	L3	L5	L3	L3	204.23
11	L3	L3	L3	L5	203.64
12	L4	L2	L2	L2	199.56
13	L4	L2	L2	L4	200.97
14	L2	L2	L4	L2	200.21
15	L2	L4	L2	L4	208.49
16	L3	L3	L1	L3	207.85
17	L2	L2	L4	L4	201.76
18	L4	L4	L4	L4	198.35
19	L4	L2	L4	L2	195.03
20	L2	L4	L2	L2	207.23
21	L3	L3	L5	L3	198.46
22	L2	L2	L2	L2	205.17
23	L2	L2	L2	L4	206.39
24	L1	L3	L3	L3	207.79

**Table 5:** A comparison among the parameters of initial and optimum designs of the composite CNG tank.

Design	Fiber Angle	Wall-Thickness of the Top and Bottom Sections (mm)	Wall-Thickness of the Middle Section (mm)	Wall-Thickness of the Main Section (mm)	Maximum Principal Stress (MPa)
Initial	17	11	4.4	3.3	206.32
Optimum	30	8.8	7.7	2.2	185.45

## Data Availability

The data presented in this study are available on request from the corresponding author.
